# Resting of Cryopreserved PBMC Does Not Generally Benefit the Performance of Antigen-Specific T Cell ELISPOT Assays

**DOI:** 10.3390/cells1030409

**Published:** 2012-07-30

**Authors:** Stefanie Kuerten, Helena Batoulis, Mascha S. Recks, Edith Karacsony, Wenji Zhang, Ramu A. Subbramanian, Paul V. Lehmann

**Affiliations:** 1 Department of Anatomy I, University of Cologne, Joseph-Stelzmann-Str. 9, 50931 Cologne, Germany; Email: helena.batoulis@uk-koeln.de (H.B.); mascha.recks@uk-koeln.de (M.S.R.); 2 Cellular Technology Ltd. (C.T.L), Shaker Heights, OH 44122, USA; Email: edith.karacsony@immunospot.com (E.K.); wenji.zhang@immunospot.com (W.Z.); ramu.subbramanian@immunospot.com (R.A.S.)

**Keywords:** cryopreservation, CEF, high-throughput, mumps, T cells

## Abstract

T cell monitoring is increasingly performed using cryopreserved PBMC. It has been suggested that resting of PBMC after thawing, that is, culturing them overnight in test medium, produces higher antigen-induced spot counts in ELISPOT assays. To evaluate the importance of overnight resting, we systematically tested cryopreserved PBMC from 25 healthy donors. CEF peptides (comprising CMV, EBV and flu antigens) were used to stimulate CD8 cells and mumps antigen to stimulate CD4 cells. The data show that resting significantly increased antigen-elicited T cell responses only for CEF high responder PBMC. The maximal gain observed was doubling of spot counts. For CEF low responders, and for mumps responders of either low- or high reactivity levels, resting had no statistically significant effect on the observed spot counts. Therefore, resting is not a generally applicable approach to improve ELISPOT assay performance, but can be recommended only for clinical subject cohorts and antigens for which it has a proven benefit. Because resting invariably leads to losing about half of the PBMC available for testing, and because doubling the PBMC numbers plated into the assay reliably doubles the antigen-induced spot counts, we suggest the latter approach as a simple and reliable alternative to resting for enhancing the performance of ELISPOT assays. Our data imply that resting is not required if PBMC were cryopreserved and thawed under conditions that minimize apoptosis of the cells. Therefore, this study should draw attention to the need to optimize freezing and thawing conditions for successful T cell work.

## 1. Introduction

Because T cells are key mediators of immunity, monitoring antigen-specific T cells has become central to progress in many fields of medical research, including infectious diseases and vaccinology [1,2,3], transplantation [4,5], allergies [6], autoimmunity [7,8,9], and tumor immunology [10,11,12]. Among the techniques available to measure T cell immunity, ELISPOT has found wide use because it is simple, reliable, sensitive, quantitative, and efficient in cell utilization [10,12,13]. 

The ELISPOT technology permits enumeration of individual antigen-specific T cells through the detection of their secretory products such as the antigen-triggered release of interferon gamma (IFN-γ). In contrast to supernatant-based assays (such as ELISA or cytokine bead arrays/Luminex), the analyte is directly captured around the secreting T cell, before it evades detection by binding to receptors of adjacent cells, dilution in the supernatant, or degradation by proteases. With a general limit of detection at 1 in 100,000 PBMC (0.001%) ELISPOT is orders of magnitudes more sensitive than supernatant-based measurements [11]. Using a similar approach, antigen-specific T cells can also be detected by flow cytometry through intracytoplasmic cytokine staining (ICS). Typical ICS is less sensitive for detecting rare cells, however, due to its detection limit of 0.02% [14]. Furthermore, ELISPOT assays detect the actually secreted analyte in pharmacologically untreated cells while ICS does not account for post transcriptional regulation and requires treatment of the cells with Golgi inhibitors to prevent secretion. 

While initially IFN-γ ELISPOT assays found wide use for the detection of Th1/Tc1 cells, continued advancement in the ELISPOT field has enabled the detection of Th2/Tc2, Th17, cytolytic, and regulatory T cells via measurements of antigen-induced release of IL-2, IL-4, IL-5 [5,15,16], IL-17 [17], Granzyme B and perforin [18,19,20], and IL-10 [21], respectively. When it comes to the detection of polyfunctional T cells, dual color ELISPOT assays permit the measurement of cytokine coexpression in individual T cells with the same sensitivity as ICS, however, low frequency responses that are difficult to detect by ICS can be readily detected by dual color ELISPOT [22]. After years of more or less successful experimentation with homemade dual color ELISPOT assays, kits recently have become readily available. These advancements have largely increased the versatility of the ELISPOT assay allowing monitoring of all major T cell effector lineages. Requiring only 100,000 PBMC per data point, ELISPOT needs less PBMC than flow cytometry-based assays which is important in the context of clinical trials where the number of PBMC that can be obtained is a critical limiting factor. This, coupled with the ease of validating and implementing ELISPOT [23], has made it one of the primary assays of choice for immune monitoring. 

The fact that antigen-specific T cells with a given specificity frequently occur at very low frequencies in PBMC continues to be a major challenge for immune monitoring. This is particularly true for chronic infections, long term immune memory, auto- and tumor-reactive T cells, and immune responses induced by subunit vaccinations. In these settings, the antigen-specific T cells can occur at or below the detection limit of even ELISPOT assays (0.001%). Therefore, one of the major goals of immune monitoring is to lower the detection limit of ELISPOT assays for antigen-specific T cells. Towards this goal, it has been reported that “overnight resting” of cryopreserved PBMC after thawing before their utilization in ELISPOT assays would increase the sensitivity of the assay, that is, provide higher antigen-induced spot counts [24,25]. Because of the promise it holds, the resting strategy has been readily embraced and recommended by some immune monitoring panels [26]. Resting, however, comes at the cost of substantial additional work, longer assay duration, and loss of PBMC that are limiting in most clinical trials. It is assumed that during the resting period apoptotic PBMC contained within the thawed PBMC die leaving a higher frequency of viable, functional cells for the assay [27]. While this rationale holds for clinical PBMC samples in which the frequency of apoptotic cells is high, does it also hold for PBMC that have been frozen according to optimized protocols when the numbers of apoptotic cells are negligible? Currently no systematic study exists that compares the benefits *vs.* downsides of overnight resting. The study reported here was designed to fill this gap. 

## 2. Results and Discussion

### 2.1. Working Hypothesis

**Figure 1:**
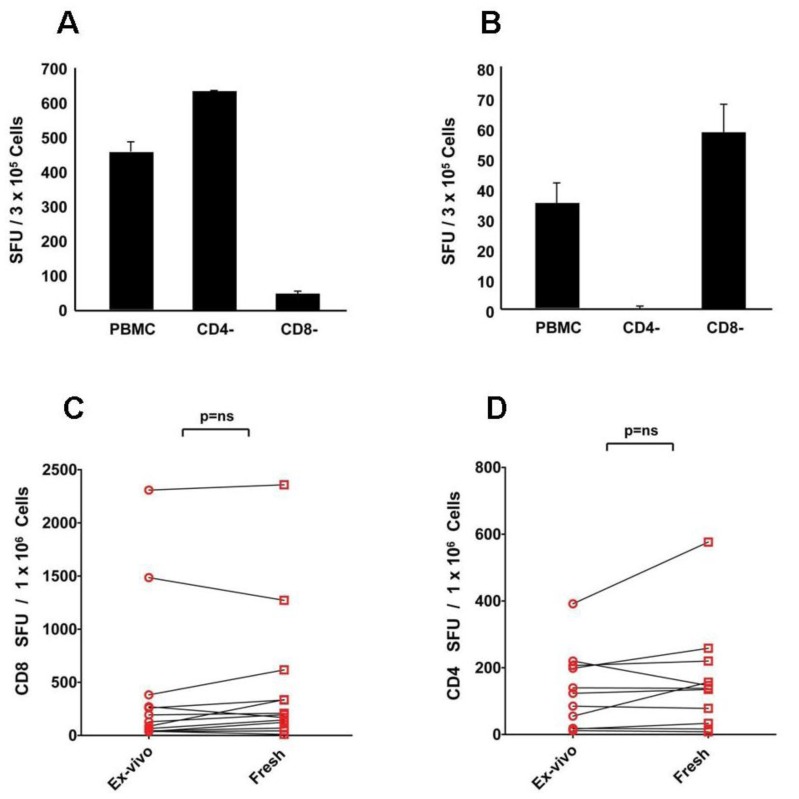
CD8 cells respond to CEF peptides (**A**), and CD4 cells to mumps antigen (**B**): these responses seen *ex vivo* are preserved after freeze thawing (**C**&**D**). To determine T cell subsets responding to the CEF and mumps antigens, magnetic affinity bead-based separation was performed depleting more than 95% of CD4 or CD8 T cells, as specified. The PBMC and cell fractions were stimulated with CEF peptides (**A**) or mumps antigen (**B**). For each condition, triplicate wells were tested with the mean and standard deviation shown. The data shown are from one of two experiments performed with similar results. Freshly isolated, non-cryopreserved PBMC (*ex vivo*) and cryopreserved PBMC that were tested directly without resting following thawing (fresh) were tested in an IFN-γ ELISPOT assay against CEF peptides (**C**) and mumps antigen (**D**). Data points obtained for individual donors are connected with a line. Each data point represents the mean of triplicate antigen-stimulated wells. Standard deviations are not shown being between 5 and 20% of the mean. For each sample, fresh or freeze-thawed, the medium background was less than 10 spots per well. Nonparametric Wilcoxon signed-rank test was used to compare matched *ex vivo vs*. fresh responses with a *p*-value ≤ 0.05 being considered significant.

Immune monitoring, in general, and ELISPOT in particular, aims at establishing accurately the frequencies of antigen-specific T cells *in vivo*. Ideally, T cell assays are performed with freshly isolated PBMC as soon as possible after the blood draw. During the 24 h duration of the ELISPOT assays antigen stimulation does not result in significant *in vitro* expansion of the antigen-specific T cells. Therefore, the frequencies measured in freshly isolated cells match the sought after *in vivo* frequency. Test results obtained with freshly isolated PBMC, therefore, can be considered as the baseline against which variations of PBMC handling, such as cryopreservation or resting, can be compared. When PBMC are cryopreserved and thawed according to protocols that we have established, the frequencies measured in freshly isolated PBMC “*ex vivo*”, and in the thawed PBMC were shown to be identical for CD4 and CD8 cells [15] which established that PBMC can be cryopreserved without loss of T cell functionality. Notably, this equal performance of non-cryopreserved “*ex vivo*” and of cryopreserved PBMC was seen without resting when the cells were plated into the assay right after thawing. In the present study, we selected CEF peptides to stimulate CD8 cells [28,29], and mumps antigen to stimulate CD4 cells. The CEF pool consists of 32 immunodominant peptides of cytomegalo-, Epstein Barr- and flu virus. Figure 1A,B shows cell separation experiments that confirm that CEF peptides activate CD8, and mumps antigen activates CD4 cells, respectively. Figure 1C,D also shows that for CEF- and mumps-antigen-elicited CD4 and CD8 cell recall responses similar IFN-γ spot counts were obtained when non-cryopreserved PBMC were tested *ex vivo* within 2 h after the blood draw, or after freeze-thawing, without resting. In this paper we refer to non-rested freeze-thawed cells as “fresh” to distinguish them from “rested” and “*ex vivo*” PBMC. In extensive subsequent studies we have confirmed for a multitude of other antigens that recall CD4 and CD8 memory cells in non-cryopreserved (“*ex vivo*”) PBMC and freeze-thawed PBMC without resting (“fresh”) provide comparable results, and we have shown that in the thawed PBMC the numbers of apoptotic cells were less than 5% when measured within 30 minutes after thawing [30]. A basic assumption of this paper therefore is that freshly thawed non-rested (“fresh”) PBMC show equal performance to non-cryopreserved *ex vivo* PBMC and can be used to test the impact of resting in ELISPOT. The basic question that we address here therefore is, does “overnight resting” reliably improve ELISPOT assay performance relative to test results obtained on freshly thawed PBMC? 

### 2.2. PBMC Donors with Low and High Responder Status to CEF Peptide Pool and Mumps Antigen

Cryopreserved PBMC of twenty five donors were randomly selected from the ePBMC donor library provided by Cellular Technology Limited (CTL), Shaker Heights, OH, USA. These PBMC were obtained from healthy donors by leucapheresis, and cells of each donor had been frozen in hundreds of identical aliquots allowing replication of experiments using the very same cell material. Hundreds of HLA-typed and immune characterized donors constitute the library. The PBMC were tested “fresh” for reactivity to CEF peptide pool, and to mumps antigen. For both antigens, the response levels ranged from undetectable (defined as the difference between spot counts in triplicate medium control wells *vs.* triplicate antigen-stimulated wells not being significantly different based on t-test evaluation) to high, reaching up to 400 antigen-elicited spots forming units (SFU) per one million PBMC over a background that was typically zero, and in no sample exceeded 10 spots per million. Because the background was negligibly low for all PBMC samples when tested “fresh” or “rested”, in the graphical representation of the data for this paper we will omit the background spots. Such a wide range of recall response levels to individual antigens within different human donors is typically seen for non-cryopreserved PBMC *ex vivo* [14,15,18,20], even when donors are vaccinated at the same time and are tested at a given time point after vaccination [18]. It is even characteristic for recall responses of inbred mice when all parameters of the immunization, the genetic background and environmental influences are kept constant [4,7,9,17]. For individual donors, the response levels for CEF and mumps were not linked: donors that displayed a high response level to CEF could be low or non-responders to mumps, and *vice versa* (examples are shown in Figure 4). In addition, donors that were low or non-responders to both antigens, responded vigorously to some others of the over 30 antigens for which all PBMC were tested (see ePBMC data base [31]—the ePBMC library consists of a large selection of precharacterized PBMC samples with established HLA types and antigen reactivities). Therefore, when low response levels were detected to either CEF peptides or mumps antigen this reflects low frequency of CEF or mumps antigen-reactive T cells in the respective PBMC donor, and not a general lack of functionality in that particular PBMC sample caused by suboptimal freezing, storage, or thawing of the cells. 

### 2.3. Resting of Cryopreserved PBMC from Low-Responders Does Not Improve Their Performance in the ELISPOT

**Figure 2:**
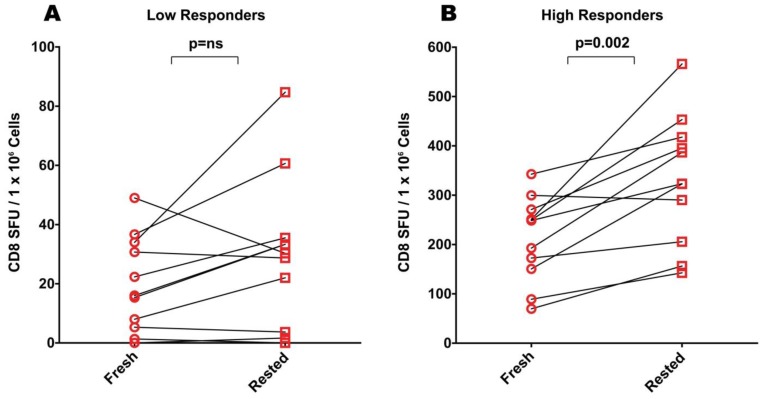
Comparison of CEF responses elicited by freshly thawed (“fresh”) and rested cryopreserved PBMC in ELISPOT assays. (**A**) CEF-specific responses among “low responders” who were defined as subjects who had fewer than 50 SFU in freshly thawed PMBC. (**B**) CEF-specific responses among “high responders” who were defined as subjects who had greater than 50 spots forming units (SFU) in freshly thawed PMBC. Data points obtained from the same donor before and after resting are connected by a line. Each data point represents the mean of triplicate antigen-stimulated wells. For each donor and antigen tested, the standard deviation was <20% of the mean value. For each sample, fresh or rested, the medium background was less than 10 spots per well. Non-parametric Wilcoxon signed-rank test was used to compare matched fresh *vs*. rested responses with a *p*-value ≤ 0.05 being considered significant.

Because antigen-specific T cells frequently occur in the low frequency range, resting of PBMC is performed in the hopes of improving the signal-to-noise performance of PBMC samples that show weak reactivity to antigen. We selected 50 antigen-induced SFU per one million PBMC as the cut-off between a low or high responder status. As shown in Figure 2A, based on SFU measured using freshly thawed PBMC, 11 of the 25 donors qualified as low responders to the CEF peptide pool. PBMC of these donors were thawed and split in two batches. One batch was plated into the ELISPOT assay within 1 h of thawing (“fresh”). The other batch was incubated for 20 h at 37 °C before plating into the ELISPOT assay (“rested”). As show in Figure 2A, six of the 11 donors displayed a slightly (approximately two- fold) elevated CEF-induced spot counts after resting. For five of the 11 donors the spot counts were decreased or equal. Overall, the difference between CEF peptide-induced SFU in fresh and rested PBMC was not significant among these low responders as evaluated by the Wilcoxon signed-rank test with a *p-*value ≤ 0.05 being considered significant. The Wilcoxon signed‑rank test is a non-parametric statistical hypothesis test used when comparing two samples, that is, it is a paired difference test.

**Figure 3:**
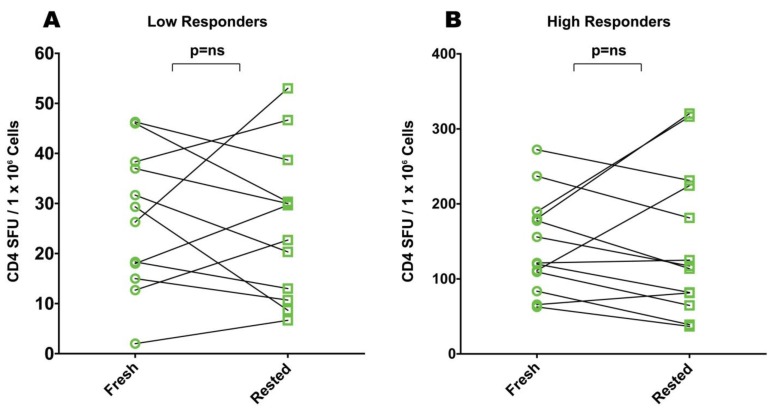
Comparison of mumps antigen elicited IFN-γ in freshly thawed and rested cryopreserved PBMC for low (**A**) and high responder subjects (**B**). Each data point represents the mean of triplicate antigen-stimulated wells. Standard deviations are not shown being between 5 and 20% of the mean. For each sample, fresh or rested, the medium background was less than 10 spots per well. Non-parametric Wilcoxon signed-rank test was used to compare matched fresh *vs*. rested responses with a *p*-value ≤ 0.05 being considered significant.

Twelve of the twenty five donors qualified as low responders for mumps antigen reactivity using the 50 SFU per million PBMC as the cut-off criterion (Figure 3A). Of these 12 low responders, the PBMC of five donors showed a slightly increased (approximately two-fold) spot count after resting. Seven showed decreased spot counts after resting. Also, for mumps antigen-induced SFU the Wilcoxon signed-rank test did not show a statistically significant difference between fresh or rested PBMC for the mumps low responders. Also the medium background was not significantly affected by resting (data not shown). 

Therefore, while one would presumably rest PBMC to obtain higher signal-to-noise performance when the frequencies of antigen-reactive T cells are low, for our low responder samples resting did not prove to provide a consistent benefit.

### 2.4. Resting of Cryopreserved PBMC from High-Responders Augments CEF-, but not Mumps Specific Responses

PBMC donors responding to antigen stimulation with more than 50 SFU per million PBMC were defined as high responders. For eight of nine CEF high responders resting resulted in increased SFU counts (Figure 2B). With the exception of one donor for whom the increase was slightly more than two fold, the increase for all other donors was less than two fold. For one of these donors, fresh- and rested PBMC provided unchanged reactivity levels. Overall, the increase seen after resting reached statistical significance as judged by the Wilcoxon signed-rank test for CEF high responders with a *p*-value of 0.002.

In measuring CD4 responses directed against mumps among the high responders, resting had no significant benefit (Figure 3B). Of thirteen mumps high responders, three showed an approximate two-fold increase in mumps-induced SFU, while for the remaining donors, the spot counts were decreased (n = 8) or were similar (n = 2). 

With the exception of CEF high responders, resting therefore had a variable and statistically insignificant effect on PBMC sample performance in ELISPOT assays. In occasional donors up to two fold increases were seen, in other donors comparable decreases in spot counts were observed. 

**Figure 4:**
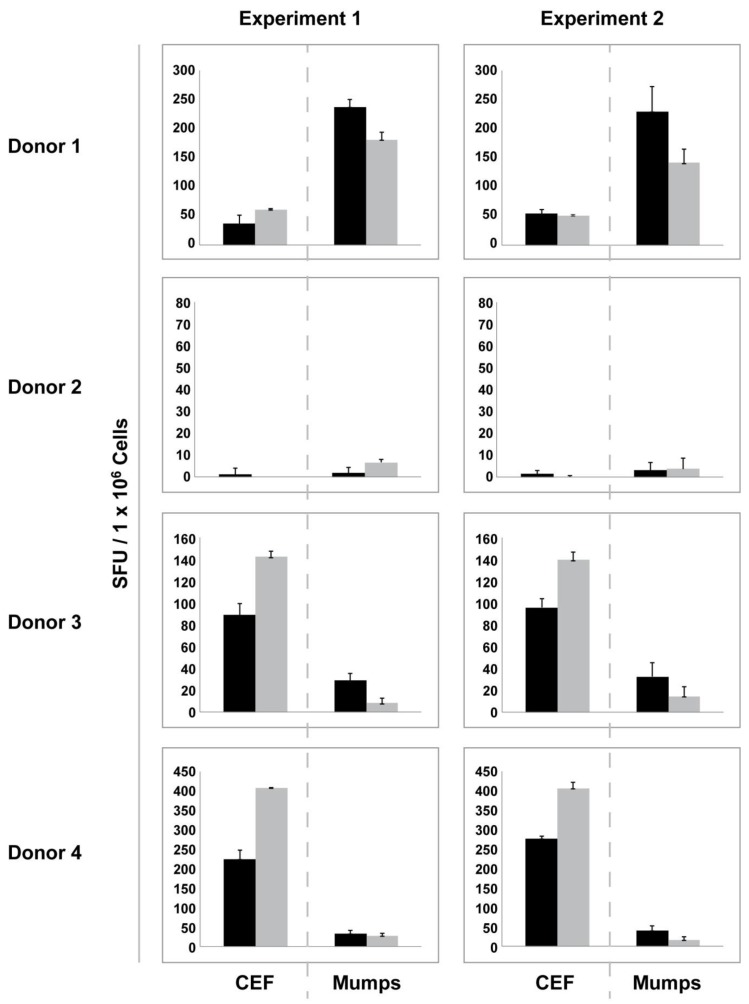
Gain or loss of function after resting is an inherent feature of PBMC samples. Responses to CEF peptide pool and mumps antigen were assessed with and without resting in two independent experiments for each donor. Data are shown for four donors who are fully representative of all the 25 donors tested. Black bars represent responses from freshly thawed cells while grey bars represent responses elicited by rested PBMC. Error bars depict the SD of the SFU from triplicate wells.

We retested the PBMC of all 25 donors with and without resting to establish whether the gain or loss that we observed after resting for a given sample is an inherent feature of a given PBMC sample, or whether samples respond unpredictably to the 20 h *in vitro* culture. In Figure 4 the results are shown for four donors that are representative of all 25 donors. First, it can be seen that a sample that is a low responder to CEF can be a high responder to mumps, and *vice versa* (donor 1 *vs.* donors 3 and 4). Donor 2, while being a low responder to both CEF and mumps is a high responder to candida and PPD (purified protein derivate) tuberculin (data not shown). Second, if a donor tested “fresh” as high or low responder to CEF or mumps in the first experiment, the same response level reproduced in the repeat experiment for both antigens. The inter-assay variation for the freshly thawed samples was not significant. Third, if after resting there was no significant gain seen for the CEF response in the first experiment, it was also not evident in the repeat experiment (donors 1 and 2), but if resting resulted in a moderate gain in the CEF response in the first experiment, a gain of similar magnitude was also seen in the repeat experiment (donors 2 and 3). Similarly, the decreased mumps response seen after resting for donors 1 and 3 reproduced in the repeat experiment, as did the unchanged mumps response levels for donors 2 and 4. Fourth, Figure 4 illustrates that the increase for one antigen after resting can result in unchanged or decreased response levels to another antigen. Fifth, this figure illustrates that in the low responder category resting does not predictably increase reactivity levels. Since increases and decreases of the antigen reactivity after resting were reproducible in the repeat experiment, they appear to be an inherent propensity of the respective PBMC.

### 2.5. Resting of PBMC Is Associated with Significant Cell Loss

**Figure 5:**
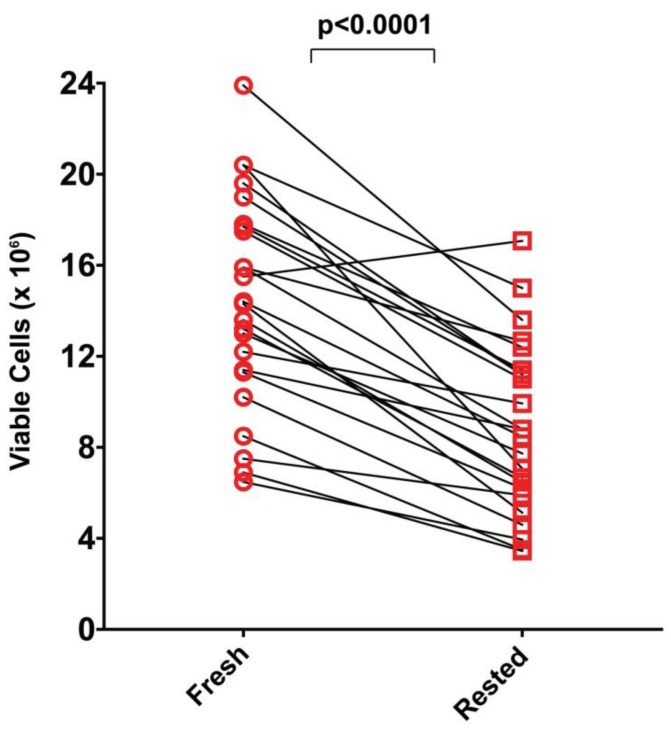
Comparison of viable PBMC recovered before and after resting. PBMC of each donor were counted by acridine orange/ethidium bromide staining within 1 h after thawing, and after a 20 h resting period. Live cell counts for each donor before and after resting are connected by a line. The data are from one experiment in which all 25 donors were tested, and are fully representative of the repeat experiment. The significance of the change was determined by the paired Wilcoxon signed-rank sum test.

Without stimulation and exposure to specific growth/survival factors most primary cells die, frequently within a short time, in cell culture. Resting PBMC in medium for 20 h represents such a stimulation-free culture period for the different cell lineages contained within PBMC. It is also conceivable that some cells/cell-lineages that appear viable directly after thawing have been injured during the freeze-thaw process and undergo apoptosis during the resting period. To establish how many cells are lost during resting, we compared viable PBMC counts within 1 h after thawing of the PBMC (“fresh”) to viable cells recovered after the 20 h resting period. Acridine orange/ethidium bromide staining was used to distinguish between live and dead cells. As shown in Figure 5, losses were seen for all but one of the 25 PBMC samples, and overall losses occurred at a similar rate, with about half of the cells lost after resting. The diminution seen was highly significant (*p* < 0.0001) as determined by paired Wilcoxon signed-rank sum test. Because less than half of the originally seeded PBMC were recovered after resting for several samples, it is advisable to plan experiments that involve rested PBMC with two to three fold excess of PBMC than what is to be plated into the assay itself. Since PBMC are limiting for most clinical studies, these losses of PBMC need to be considered *vs.* possible benefits of assay results obtained through resting. 

### 2.6. Frequencies of CD4 and CD8 Cells Are Unchanged between Fresh and Rested PBMC

For CEF high-responders, we observed in rested samples increased frequencies of CD8 antigen-specific responses (see above, Figure 2B), which could partly explain published claims [24,25]. This finding could be explained by a preferential survival of CD8 cells in such PBMC samples over the resting period while other cell types could have died more rapidly leading to a CD8 cell enrichment in the rested sample. Increased responses to CEF in conjunction with decreased mumps responses after resting (e.g., donor 3 in Figure 4) might result from more rapid dying off of CD4 cells. To formally test this hypothesis, we used flow cytometry to monitor potential changes in the frequency of CD4^+^ and CD8^+^ T cell lineages following resting. 

**Figure 6:**
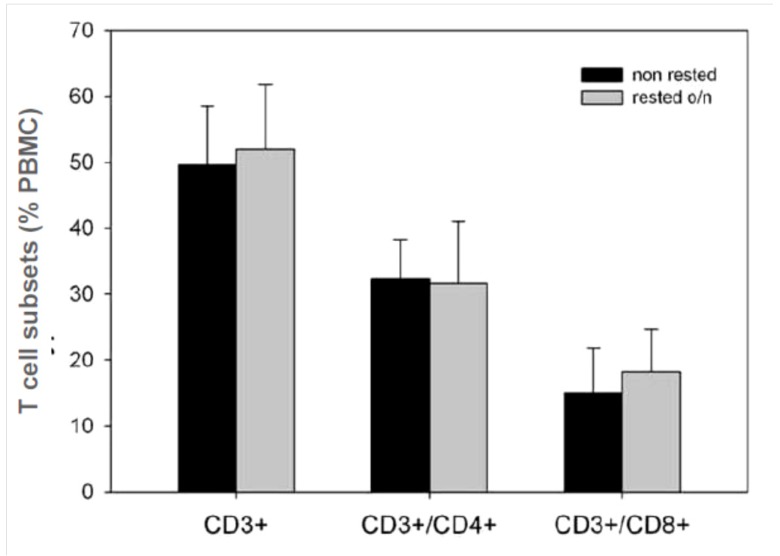
Frequencies of T cells and the CD4/CD8 subpopulations in PBMC before and after 20 h resting determined by flow cytometry. For all 25 donors, the percentages of CD3^+^, CD3^+^/CD4^+^, and CD3^+^/CD8^+^ cells were established once before (black bars), and once after (grey bars), resting. The mean and SD for the 25 PBMC of both groups are shown.

Phenotypic characterization of the freshly thawed and rested PBMC by flow cytometry did not show any statistically significant changes in CD8^+^ or CD4^+^ T cell subsets due to resting (Figure 6). Importantly, no correlation existed between the moderate changes in the percentage of CD8^+^ T cells in a given PBMC sample and the CEF peptide-elicited SFU response; similarly, slight increases/decreases in frequencies of CD4 cells after resting did not result in a proportional increase/decrease in the mumps recall response (data not shown). The changed T cell responses seen after resting, therefore, resulted from changes in T cell functionality, not from altered T cell frequencies within the tested sample.

### 2.7. Increasing the PBMC Numbers Plated in ELISPOT, Unlike Resting, Predictably Increases the Signal-to-Noise Performance of the Assay

In spite of the increased work load and the cell losses, resting has been advocated because it is presumed to improve the signal-to-noise performance of ELISPOT assays. As shown above, resting does not predictably have such an impact on low responders. Worse, for the low responders, rested samples could provide decreased antigen-induced spot counts, in particular for CD4 cells (e.g., donor 3 in Figure 4). 

**Figure 7:**
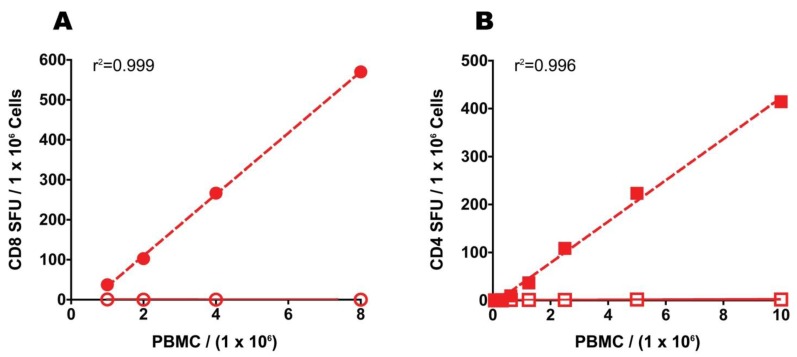
Relationship between the numbers of freshly thawed PBMC added per well and observed SFU in ELISPOT. CEF peptide (**A**) or mumps antigen-elicited SFU (**B**) plotted against the number of PBMC added per well. The PBMC were plated in serial dilution with antigen (closed symbols) or without (medium alone, open symbols) keeping all other assay variables constant. The regression for antigen-induced SFU was calculated and is shown. The data are from one experiment.

Due to the unpredictable outcome of resting, and the need for two to three times more PBMC to accommodate the cell loss, we tested the hypothesis that the desired increase in signal-to-noise performance of the PBMC can be accomplished simply by increasing the numbers of freshly thawed PBMC plated into the ELISPOT assay. To evaluate this hypothesis directly, we plated unrested PBMC in two-fold serial dilutions and asked if observed CEF and mumps antigen-specific SFU show a linear increase proportional to the number of cells assayed. The data presented in Figure 7 suggest that in the 0.1 to 0.8 million PBMC per well range there is a high degree of linearity between the numbers of PBMC plated and the observed SFU both in the CD8 (Panel A) and CD4 (Panel B) compartments. This linear relationship between spot counts and PBMC numbers (in the range of 100,000–800,000 cells/well) was confirmed in nine independent experiments using different antigens and PBMC of different donors (data not shown). Therefore, by doubling or tripling the numbers of freshly thawed PBMC plated into an ELISPOT assay one can predictably accomplish what resting does not, and in spite of the increased cell numbers needed, this strategy does not require more cells than resting does. 

Our literature research has identified only two original studies in which a beneficial effect of resting has been described directly. In one study [24], fresh and rested PBMC of four patients with Stage III and IV follicular lymphoma were compared; in addition to bearing a large tumor burden, these patients were under chemotherapy. In the second study [25], data are shown for four healthy donors, who were assessed for responses to CEF and CMV. Of these, only one (D4) was in the low responder category by our definition—no benefit of resting was seen for this donor. The remaining three donors were CEF or CMV high responders by our definition—an approximately two-fold increase was seen in two high responders. Janetzki *et al.* [25] also refer to a study by Kierstead *et al*. that suggests overnight resting is essential to achieve optimal T cell responses comparable to those from unfrozen PBMC though details of the comparison experiments are not provided and the data are listed as “not shown” in the study [32]. In another report resting was positively correlated with the detection of low frequency CD8 cells [26], but this study was inconclusive about resting itself because resting was just one of several assay parameters in which the compared results differed. Increasing the numbers of cells plated was another assay parameter in which the results differed, which in itself suffices to predictably improve the signal to noise performance of the assay (Figure 7 in our study). Overall, in that study PBMC of 7 donors were tested for reactivity to CMV and flu peptides of which three possessed CMV-specific CD8 cells, and five responded to the flu peptides. Results were compared between thirteen centers in which “ELISPOT analysis was performed according to 11 more or less different protocols”, and the influence of different parameters on the number of positive responses was studied. 

In our study presented here, the benefit of resting was tested systematically in a larger cohort of 25 subjects while maintaining all other assay parameters constant. Resting caused a predictable gain in assay sensitivity for CD8 responses of high-magnitude alone. This gain was on average less than two-fold, and while statistically significant, it might have limited significance in immune diagnostic terms as the responses that are enhanced through resting are already clear-cut without resting. Also, the added labor and increased need for sample material might not be justified. Importantly, the responses seen with freshly isolated (non-cryopreserved) PBMC correspond with responses seen with freshly thawed, unrested PBMC (Figure 1 C,D). Therefore the enhancement obscures the sought after *ex vivo* frequencies of antigen-reactive T cells. For low magnitude CD8 responses, and for CD4 reactivity of all magnitudes, resting had an unpredictable effect: decreased or unchanged reactivity was seen just as frequently as instances of increased reactivity. In contrast, doubling the number of freshly thawed PBMC predictably doubles the signal-to-noise performance of ELISPOT assays. Therefore, by doubling the number of freshly thawed PBMC plated in an ELISPOT assay one can reliably accomplish accurate monitoring of both high- and low magnitude responses derived from CD8 and CD4 cells alike, with less labor, and with the same amount of cell material. 

Our studies were done with PBMC of healthy donors that were cryopreserved, importantly, according to optimized protocols that permit full recovery of CD4 and CD8 T cell reactivity after optimal thawing, as we have previously shown [15] and as confirmed in Figure 1 A,B for the CEF and mumps responses that we have studied in this paper. We can envision several scenarios in which resting benefits T cell monitoring. 

Resting is likely to benefit T cell monitoring if the number of apoptotic cells is high in thawed samples. The number of apoptotic cells might be high if the PBMC were not frozen or thawed under optimized conditions. In this situation resting might help to rescue a result, but for successful T cell work attention should be drawn to optimized freezing and thawing conditions. Following such protocols, PBMC of healthy donors should yield more than 95% viable, and less than 5% apoptotic/dead cells. It will be important for the field to adopt optimized cryopeservation/thawing conditions. 

Resting might also be beneficial for PBMC of subjects with certain diseases, or receiving medication/treatments that increases apoptosis of T cells or APC. The PBMC of the patients with Stage III and IV follicular lymphoma that in addition received chemotherapy [24] are likely to fall into this category. Furthermore, the numbers of apoptotic cells can be elevated if the samples were shipped under suboptimal conditions or requiring long transit periods, or were stored under suboptimal conditions before freezing. In our experience, human PBMC should never be chilled but shipped (as heparinized full blood) and processed (washed) warm. If apoptotic cells are present in PBMC in significant numbers, these, being still alive, will be stained and counted as live cells, but they will be dead by the time the functional assay is performed. Therefore, without accounting for apoptotic cells, one would plate fewer functional PBMC than the live cell count suggests, and the frequency of SFU will be accordingly decreased. After resting, with the apoptotic population gone, the relative frequency of functional T cells will be increased, and subsequently also the spot count. Apoptotic cells can be selectively stained by dyes such as Jo-Pro. Therefore, we support the CIMT (Association for Cancer Immunotherapy) suggestion to include counting of apoptotic cells into the immune monitoring routine [26].

Presently it is not well established whether apoptotic cells are just non-functional bystander cells, or whether they exert inhibitory effects in functional T cell assays in addition. If they are passive, adjusting the counts of freshly thawed cells to functional cells (viable minus apoptotic) might accomplish in a simpler way what resting does. If apoptotic cells are inhibitory, however, selective depletion of such cells or resting will be required to improve the performance of PBMC samples that contain significant numbers of apoptotic cells. Unlike necrosis that triggers inflammation, apoptosis is an intrinsically non-phlogistic process [33]. Apoptotic cells can suppress proinflammatory responses of neutrophils and mononuclear phagocytes, and can elicit an anti-inflammatory response by these cells [34,35]. The active secretion of lactoferrin by cells undergoing apoptosis is one of the key mechanisms by which apoptotic cells instruct the innate immune system [36]. Lactoferrin binds to receptors on phagocytes and inhibits their pro-inflammatory responses via NF-κB [37]. Thus, lactoferrin can inhibit production of pro-inflammatory mediators such as TNF-α and IL-6 [37,38], and can induce the secretion of anti-inflammatory cytokines including TGF-β, IL-4 and IL-10 [39,40]. It will need to be established experimentally whether and how the presence of cells undergoing apoptosis in PBMC affect T cells in functional assays such as ELISPOT or ICS. 

Resting might also benefit T cell monitoring if the serum of the donor contains immune suppressive factors or drugs, for example in cancer patients or HIV-infected subjects. When isolated from such donor’s own serum, and cultured overnight in a neutral medium, such PBMC might recover functionality. Similarly, resting might be of benefit if the serum contains cytokines that stimulate cells of the innate immune system to produce the analyte. For IFN-γ assays, for example, IL-2 is a potent stimulator of IFN-γ production in macrophages, NK and dendritic cells and several other lineages. In such cases, the background in the medium control will be high. IFN-γ spots produced by antigen‑stimulated T cells are typically larger than IFN-γ spots produced by cells of the innate immune system and therefore can be identified by proper gating strategies during ELISPOT analysis. Because several of the above cell lineages of the innate system are plastic adherent, these will be depleted during the resting step, resulting in a lower background. Additionally, the stimulatory effect of the serum cytokines that acted *in vivo* will wane during resting in neutral test medium *in vitro*. 

## 3. Experimental Section

### 3.1. Thawing and Resting of Cryopreserved PBMC

Cryopreserved human PBMC of 25 donors were acquired from a library of PBMC (ePBMC, CTL‑CP1) offered by Cellular Technologies Ltd. (CTL, Shaker Heights, OH, USA). In addition to high-resolution HLA-typing, these PBMC had been previously characterized for T cell reactivity to a panel of antigens. Different aliquots of the same PBMC sample permit the exact reproduction of these reactivities in subsequent experiments. Therefore, these pre-established response levels can serve as reference values (while also serving as positive controls), against which unknown responses of a given PBMC sample can be measured. Polyclonal stimulation with SEB or PHA can also serve as such a reference standard/positive control, but for measuring antigen-specific T cell responses, protein or peptide recall antigens provide more relevant reference values. Also, the spot morphology induced by polyclonal stimulators is fundamentally different from spots elicited by antigens. 

Prior to testing, the PBMC cryovials stored in liquid N_2_ vapor phase were transferred to dry ice in styrofoam containers for transport to and short term storage in the laboratory. The cells were thawed following a protocol that we have established to provide the optimal recovery and functionality for cryopreserved PBMC [41]. Specifically, to rapidly warm up to 37 °C, the cryovials were placed for 8 minutes into a 37 °C bead bath (CTL-BB-001). The cryovials were inverted twice to resuspend the cells, and the 1 mL cell suspension contained in each cryovial (10 million cells) was gently aspirated utilizing a wide-bore 2 mL pipette and transferred into a 15 mL V bottom Falcon tube. To recover the residual cells, the cryovials were rinsed by adding 1 mL 37 °C warm CTL Anti-Aggregate Wash™ Medium (CTL-AA-005) that contains benzonase. Additional 8 mL CTL Anti-Aggregate Wash™ Medium at 37 °C were added to the 15 mL tube at a rate of 2 mL per 5 seconds. Before washing, an aliquot of these PBMC was counted by fluorescence microscopy using acridine orange and ethidium bromide (AO/EB) to stain live and dead cells respectively. Apoptotic cells were stained with Jo-Pro (Invitrogen) and counted similarly. PBMC were washed twice in 10 mL CTL-Test™ Medium (CTLT-005) and resuspended at a final concentration of 3 × 10^6^ PBMC/mL in the same medium. When testing the freshly thawed PBMC (we refer to such cells as “fresh” in this paper), these cells were plated onto an ELISPOT assay plate within 1 h of thawing. For resting, cells were incubated for 20 h at 37 °C at a concentration of 3 × 10^6^ cells/mL in CTL-Test Medium, washed twice in CTL-Test Medium and readjusted to 3 × 10^6^ cells/mL in this medium before plating into the ELISPOT assay.

### 3.2. Human Interferon-γ ELISPOT Assay

Human Interferon-γ ImmunoSpot kits (CTL-H1FNG-1/5M) were obtained from CTL. The assay was performed according to the manufacturer’s recommendations. The PVDF membranes were not pre-wetted with ethanol as it is not required nor recommended for this kit. The antigens, CEF peptide pool (CTL-CEF-002; 2 µg/mL) or mumps antigen (BioWhittaker, Walkersville, MD, USA, Lot# IV0094, 1:80 dilution) were plated first in triplicates to the capture antibody pre-coated assay plate in a final volume of 100 µL per well—the antigens were dissolved in CTL Test Medium (CTLT-005) which also constituted the negative/medium control. These plates containing the antigens and medium control were stored at 37 °C in a CO_2_ incubator until the cells were ready for plating. The thawed PBMC were adjusted in CTL Test Medium to 3 million PBMC/mL of which 100 µL/well (300,000 cells/well) were plated per well using wide-bore pipette tips. Plates were gently tapped on each side to ensure even distribution of the cells as they settle, and incubated for 24 h at 37 °C in a CO_2_ incubator. Following completion of the ELISPOT protocol, the plates were air dried in a laminar flow hood prior to analysis. 

ELISPOT plates were scanned and analyzed using an ImmunoSpot S6 Core Reader by CTL. Spot Forming Units (SFU) were automatically calculated by the ImmunoSpot Software for each antigen stimulation condition and the medium (negative) control using the SmartCount™ and Autogate™ functions [42]. Data are presented as mean SFU per 10^6^ PBMC induced by the specified antigen minus the SFU count in the negative control. In all experiments, the negative control was less than 10 SFU per 10^6^ PBMC.

### 3.3. Flow Cytometry

Total CD3^+^; CD3^+^/CD4^+^, and CD3^+^/CD8^+^ T cell subsets were assessed by flow cytometry. Briefly, 500,000 cells were stained in 5 mL Falcon round-bottom tubes with human anti-CD3^FITC^ (clone HIT3a), and CD4^PE^ (clone RPA-T4), or anti-CD8^PE^ (clone RPA-T8) (all BD Biosciences, San Jose, CA, USA) and fixed in 2% formaldehyde. A minimum of 200,000 events were acquired using BD FACSCanto III (BD) and analyzed with *FACSDiva^TM^*, version 4 software [43] (BD). 

### 3.4. Statistics

The relationships between SFU and cell numbers were tested by linear regression. The comparisons of matched pre- and post-thawing SFU were tested by a two-tailed, non-parametric Wilcoxon signed-rank test. A *p*-value of 0.05 or less (two-tailed) was considered significant. 

## 4. Conclusions

Our data clearly show that resting is not a universally applicable approach to improve the performance of cryopreserved PBMC for detection of low frequency T cell responses. In contrast, considering that more than half of the cells are lost during resting, one can reliably double the signal to noise performance of T cell assays by doubling the number of freshly thawed cells plated into the assay, that is, using the amount of cells that resting would require. Doubling cell numbers when possible can both be a convenient and more accurate measure of T cell responses. While resting might be beneficial for special clinical conditions, the data presented here suggest it is clearly not advantageous or accurate in monitoring immune responses among healthy subjects, and might not be precise for samples derived from many disease states. From the unpredictable increase or decrease of T cell reactivity after resting relative to the T cell frequencies *ex vivo*, to potentially missing positive responses due to substantial cell losses, to the added labor and complexity of the protocol, investigators are well advised to establish whether resting indeed will benefit immune monitoring in their particular study population. 
